# Efficient Epidermal Growth Factor Receptor Targeting Oligonucleotide as a Potential Molecule for Targeted Cancer Therapy

**DOI:** 10.3390/ijms20194700

**Published:** 2019-09-22

**Authors:** Tao Wang, Svetlana Philippovich, Jun Mao, Rakesh N. Veedu

**Affiliations:** 1 Centre for Molecular Medicine and Innovative Therapeutics, Murdoch University, Perth 6150, Australia; Tao.Wang@murdoch.edu.au (T.W.); 32351588@student.murdoch.edu.au (S.P.); 2 Perron Institute for Neurological and Translational Science, Perth 6009, Australia; 3 College of Basic Medical Sciences, Dalian Medical University, Dalian 116044, China; maojun1116@163.com

**Keywords:** Chemically-modified oligonucleotides, nucleic acids, aptamers

## Abstract

Epidermal growth factor receptor (EGFR) is associated with the progression of a wide range of cancers including breast, glioma, lung, and liver cancer. The observation that EGFR inhibition can limit the growth of EGFR positive cancers has led to the development of various EGFR inhibitors including monoclonal antibodies and small-molecule inhibitors. However, the reported toxicity and drug resistance greatly compromised the clinical outcome of such inhibitors. As a type of chemical antibodies, nucleic acid aptamer provides an opportunity to overcome the obstacles faced by current EGFR inhibitors. In this study, we have developed and investigated the therapeutic potential of a 27mer aptamer CL-4RNV616 containing 2′-O-Methyl RNA and DNA nucleotides. Our results showed that CL-4RNV616 not only displayed enhanced stability in human serum, but also effectively recognized and inhibited the proliferation of EGFR positive Huh-7 liver cancer, MDA-MB-231 breast cancer, and U87MG glioblastoma cells, with an IC50 value of 258.9 nM, 413.7 nM, and 567.9 nM, respectively. Furthermore, TUNEL apoptosis assay revealed that CL-4RNV616 efficiently induced apoptosis of cancer cells. In addition, clinical breast cancer biopsy-based immunostaining assay demonstrated that CL-4RNV616 had a comparable detection efficacy for EGFR positive breast cancer with commonly used commercial antibodies. Based on the results, we firmly believe that CL-4RNV616 could be useful in the development of targeted cancer therapeutics and diagnostics.

## 1. Introduction

The epidermal growth factor receptor (EGFR) is a subfamily of four closely related receptors including EGFR (ErbB-1), HER2/neu (ErbB-2), Her 3 (ErbB-3), and Her 4 (ErbB-4) [1]. As one of the most investigated tyrosine kinases, EGFR over-expression has long been associated with the development of a number of cancers and believed to be a negative cancer prognostic factor [2]. Consequently, neutralization of EGFR signaling by blocking EGFR binding sites on the extracellular domain represents a plausible strategy to prevent EGFR-expressing tumor growth. Over the past decades, various EGFR inhibitors (erlotinib hydrochloride, cetuximab, and necitumumab) [3], have been approved for cancer treatment. However, the application of these EGFR inhibitors has been compromised by toxicity and acquired drug resistance [4], which demonstrated the need for developing innovative therapeutic molecule capable of overcoming the challenges faced by existing EGFR inhibitors. 

Over the past decades, nucleic acid aptamers (also known as chemical antibodies) [5,6] have emerged as a promising approach for precision cancer therapeutics and diagnosis [7]. Aptamers are short single-stranded oligonucleotides that can bind to a target ranging from small molecules to complex proteins with high specificity and affinity because of their ability to adopt three-dimensional shapes in solution [5,6,8]. Compared to antibodies, aptamers certainly possess advantages such as easy laboratory production, high stability, low or no immunogenicity, ability to reverse target binding using a complementary sequence, freedom to incorporate multiple chemical modifications without affecting the targeting affinity or specificity, and low cost. Consequently, over the past decades, aptamer-based nanotechnology has been comprehensively investigated for biosensor development [9,10] and targeted therapeutics to deliver various types of cargoes ranging from small molecule drugs [11,12], nucleic acids [13] to nano-sized extracellular vesicles [14,15]. Recently, a 39mer EGFR targeting RNA aptamer (CL4) has been developed [16]. As current EGFR inhibitors are associated with different drug resistance mechanisms, EGFR aptamer may prove useful in the treatment of cancers that are resistant to current EGFR inhibitors. However, while exploring the scope of CL4 aptamer in our research, we found that this reported aptamer degraded in human serum which will impede its further clinical translation. In addition, generation of shorter aptamer sequences, in line with the FDA approved 27mer Macugen, could also reduce the cost and toxicity. To address these limitations, we envisioned the development of a robust EGFR aptamer by rationale truncation and chemical modifications of the existing 39mer CL4 and extend its potential by evaluating in various cancer cells including malignant glioblastoma cells. 

## 2. Results and Discussion

### 2.1. Truncation and Chemical Modification of EGFR Aptamer

The binding property and biological functions of an aptamer sequence rely on its unique structure. However, only certain distinct regions are responsible for target binding. Therefore, rational truncation of aptamers can in some cases yield shorter aptamer sequences without losing their affinity and target specificity [7]. In line with previous reports, we have used the Mfold and RNAfold web server platforms [17,18] to predict and analyze the secondary structure of CL-4 aptamer. We hypothesized that the 13 nucleotides tail region located at the 5′-end of the sequence (Figure S1) may not participate and influence on EGFR binding, which led us to truncate the 39mer CL-4 aptamer to new 27mer variants (Table 1). However, it needs to be kept in mind that the 3-D structural conformation of a single-stranded DNA or RNA aptamer sequence will be largely affected by the adjacent nucleotide sequences, and truncation can lead to structural changes which could also influence the binding properties. Therefore, the binding affinity of a truncated sequence has to be tested experimentally to make sure that the deletion of adjacent sequences does not really affect its tertiary structure and the binding property. 

The original CL-4 aptamer is a 2′-Fluoro pyrimidine-modified RNA sequence. Although 2′-Fluoro pyrimidine modification has been explored previously and contributed to the clinical development of Macugen (the FDA approved aptamer drug [19]), a recent in vivo study showed that the oligonucleotides with 2′-Fluoro modification may cause substantial toxicity after systematic administration [20,21]. Unlike Macugen which meets its target by topical intravitreal injection, systemic administration is indispensable for anti-cancer drug development, especially for the treatment of metastatic cancers. Towards this goal, the serum stability of the developed EGFR aptamers needs to be particularly addressed. According to our initial test, the original CL-4 aptamer displayed substantially weaker stability in 90% serum, making it not suitable for further in vivo development. To address these limitations, we introduced 2′-*O*-Methyl RNA (2′-OMe) modification to improve the stability of the developed aptamers. 2′-OMe endows oligonucleotides with superior immune compatibility, minimized toxicity as well as enhanced stability, and it is worth mentioning that it has been identified in various endogenous nucleic acid molecules such as ribosomal RNA, small nuclear RNA, and spliceosome in physical conditions [22]. As demonstrated in Table 1, two different 2′-OMe-modified truncated 27mer aptamers were designed and synthesized including CL-4RNV615, a full-length 2′-OMe modified variant, and CL-4RNV616, a 27mer DNA-A modified 2′-OMe variant, in which all ‘A’ nucleotides were modified with DNA-A nucleotides. In addition, we also designed and synthesized the corresponding 27mer full RNA-modified variant (CL-4RNV617) and full DNA modified variant (CL-4RNV618). 

### 2.2. CL-4RNV616 Specifically Recognized Recombinant EGFR Protein with Enhanced Serum Stability

**Figure 1:**
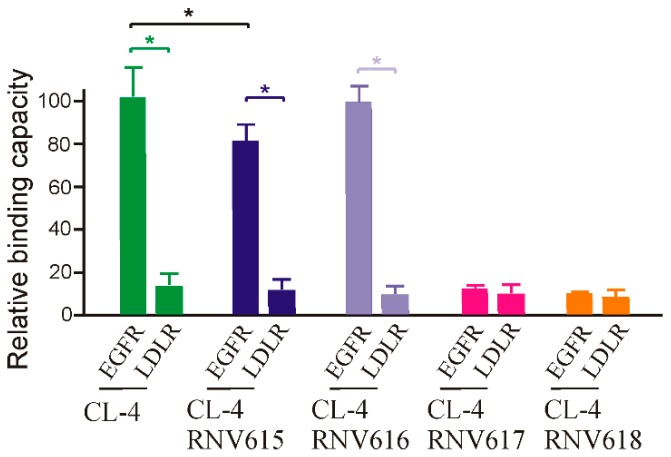
Relative binding capacity of different modified EGFR aptamer sequences and their specificity analysis using a negative target of the lipoprotein receptor protein (LDL-R) protein. EGFR protein was incubated with 100 nM of biotin labelled aptamers CL-4RNV615 to CL-4RNV618 for 1 h, and the binding capacity was analysed by ELONA assay. * *p* < 0.05.

Firstly, we performed the binding affinity screening of the developed aptamers to human recombinant EGFR protein via ELONA assay using procedures similar to the previous report [23]. In addition, we also used a negative control receptor protein, low-density lipoprotein receptor (LDL-R), to verify any non-specific interaction. As shown in Figure 1, our initial analysis found that CL-4, CL-4RNV616, and CL-4RNV615 showed high binding affinity to EGFR protein, and as expected all screened aptamer candidates showed negligible binding to the negative control LDL-R protein, which is indicative of target-specific binding of CL-4, CL-4RNV615, and CL-4RNV616. Importantly, although significant difference as defined as *p* < 0.05 was not observed between CL-4RNV615 and CL-RNV616 groups for EGFR binding, the original CL-4 aptamer demonstrated higher binding capacity (*p* < 0.05) than CL-4RNV615, but not CL-4RNV616. We, therefore, suggested that the DNA-A modified CL-4RNV616 aptamer displayed better binding compared with the full 2′-OMe-modified CL-4RNV615 aptamer (Figure 1). In contrast, the full RNA-modified CL-4RNV617 and full DNA-modified CL-4RNV618 failed to demonstrate any specific binding to EGFR protein (Figure 1). As CL-4RNV616 was found to be comparatively better in targeting EGFR, we then used this 2′-OMe/DNA-A mixmer EGFR aptamer for further analysis. 

**Figure 2:**
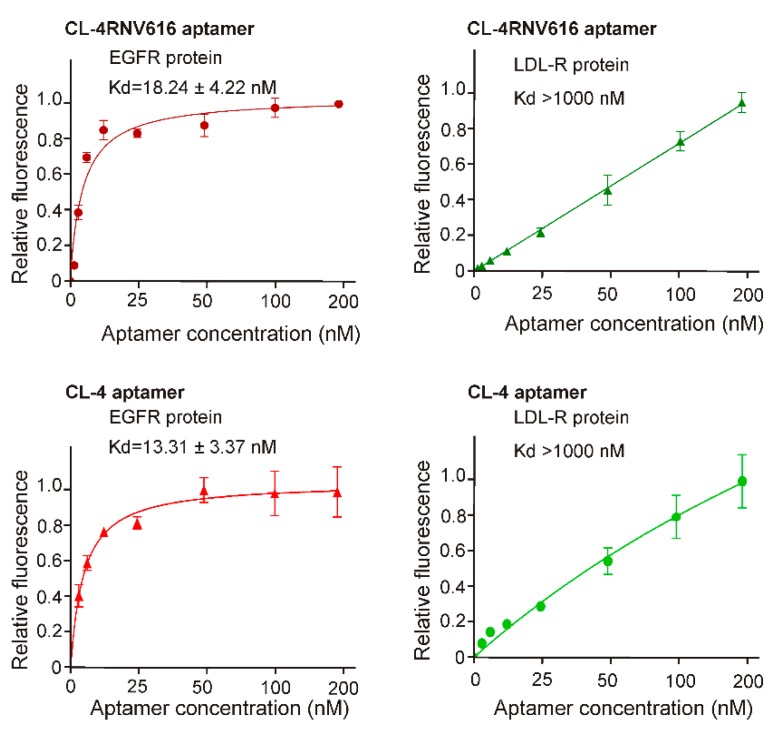
Determination of binding affinity of CL-4 and CL-4RNV616 aptamers to EGFR protein. Equilibrium dissociation constants (*K*_d_) was determined by incubating EGFR protein at varying concentrations of aptamer (0–200 nM) using LDL-R protein as a negative control. *K*_d_ was derived using the GraphPad Prism program 8.0.

**Figure 3:**
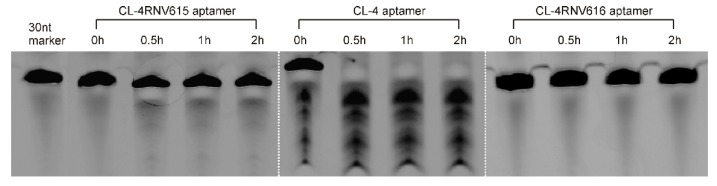
Evaluation of the stability of aptamers in 90% human serum. After incubation, samples were separated on a 20% Urea-PAGE denaturing gel, with a 30nt RNA sequence as the loading control.

To gain further insights on dissociation constant, ELONA assay was repeated using multiple concentrations (200 nM, 100 nM, 50 nM, 25 nM, 12.5 nM, 6.25 nM, 3.125 nM) of CL-4RNV616 and CL-4. CL-4RNV616 showed a dissociation equilibrium constant (*K*_d_) of 18.24 nM to EGFR protein, which is comparable with the original CL-4 aptamer with a *K*_d_ value of 13.31 nM (Figure 2). At the same time, we have also performed the dissociation constant of CL-4RNV616 and CL-4 aptamer using a negative protein target, LDL-R, to analyse the specificity. As expected, both CL-4RNV616 and CL-4 aptamers did not show any noticeable binding to LDL-R protein (*K*_d_ >1000 nM), demonstrating higher target specificity. We then analysed the stability of CL-4, CL-4RNV616 and the full 2′-OMe-modified aptamer CL-4RNV615 in 90% human serum over a period of 2 hrs. As displayed in Figure 3, the results showed that both CL-4RNV615 and CL-4RNV616 were found to be very stable even after 2h of incubation, whereas the control CL-4 aptamer degraded after 30 min of incubation. 

### 2.3. CL-4RNV616 Aptamer Specifically Recognized EGFR Protein in Human Cancer Cells 

**Figure 4:**
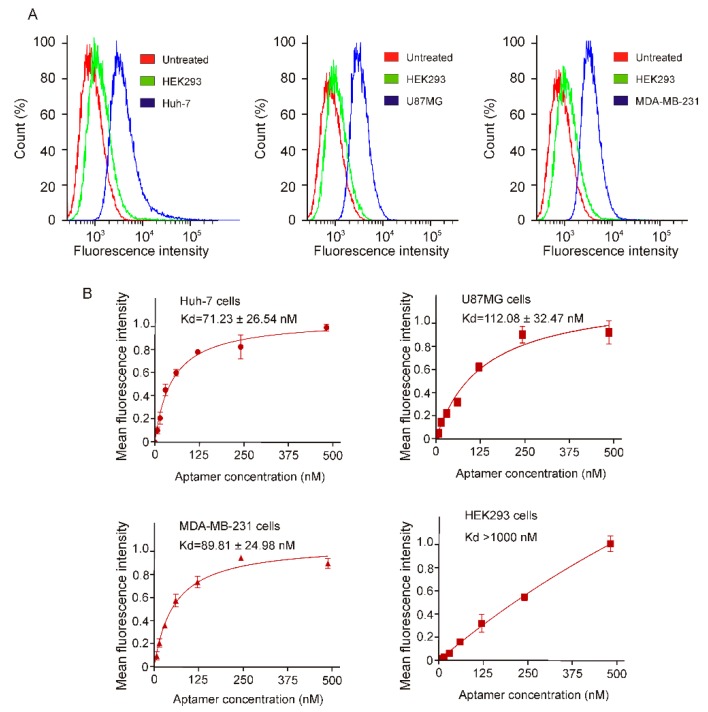
Determination of specific binding of CL-4RNV616 aptamer to EGFR positive cells via FACS assay. (**A**) Quantification of the binding of CL-4RNV616 aptamer to EGFR-positive cell lines and to the EGFR-negative HEK-293 cells via flow cytometric analysis. FAM labelled CL-4RNV616 aptamer was incubated at a concentration of 200 nM with EGFR positive or negative cell lines for 30 min at 37 °C. (**B**) Determination of equilibrium dissociation constants (*K*_d_) of CL-4RNV616 aptamer to EGFR positive cells via flow cytometry by incubating cells at varying concentrations of aptamer (0–500 nM) using an EGFR negative HEK293 cell line as a negative control. *K*_d_ was derived using the GraphPad Prism program 8.0.

For targeted cancer therapy, an aptamer has to recognize its protein targets in their native state on living cancer cell surface and efficiently internalize upon aptamer-target binding [24]. Towards this goal, next, we analyzed the binding potential of CL-4RNV616 aptamer using different types of cancer cells. After incubating CL-4RNV616 with both EGFR-positive MDA-MB-231 breast cancer cells, Huh-7 liver cancer cells, U87MG glioma and EGFR-negative HEK293 cells at 37 °C for 30 min, the binding specificity was then investigated via flow cytometry assays. As shown in Figure 4A, the results clearly demonstrated that CL-4RNV616 could efficiently bind to all of the three tested EGFR-positive cell groups by displaying an excellent shift in fluorescence signals. Further binding affinity experiments revealed that the CL-4RNV616 aptamer has a medium-high binding capacity to EGFR protein on their natural cell surface state, with dissociation equilibrium constant of 71.23 nM for Huh-7, 112.08 nM for U87MG, and 89.81 nM for MDA-MB-231 respectively (Figure 4B). However, CL-4RNV616 aptamer did not show any noticeable binding to the EGFR negative HEK293 cells (*K*_d_ >1000 nM) demonstrating its target specificity (Figure 4B). 

**Figure 5:**
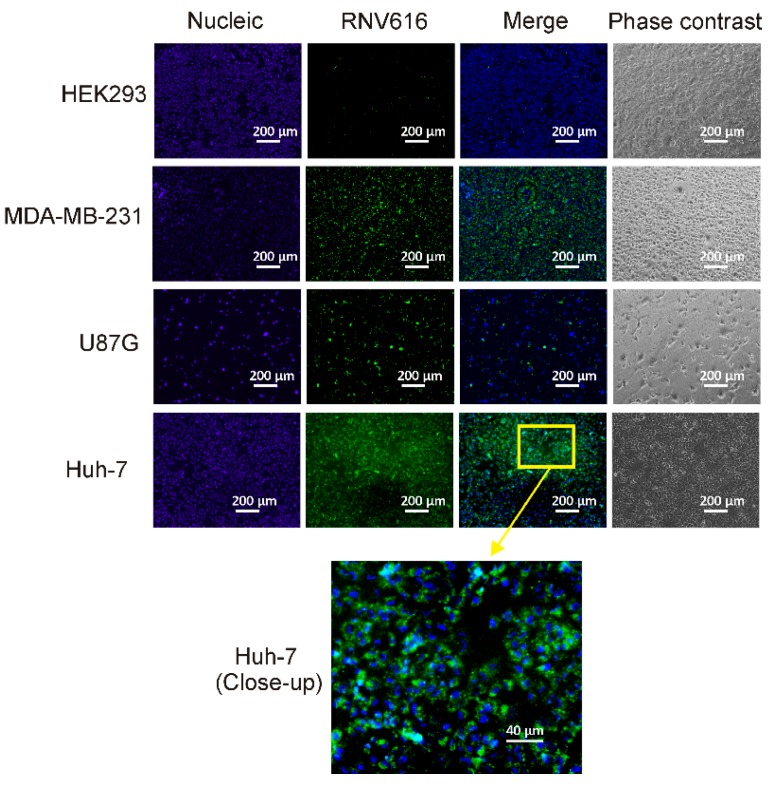
Specific binding of CL-4RNV616 aptamer to EGFR-positive cells. CL-4RNV616 aptamer specifically binds to EGFR-positive Huh-7, U87MG, and MDA-MB-231 cells but not to EGFR-negative HEK293 cells. Cells were imaged via fluorescent microscopy after incubation with 200 nM of aptamer at 37 °C for 30 min. Green, FAM (aptamer), and blue, Hoechst 33342 (nuclei).

The medium-high binding affinity of the CL-4RNV616 aptamer to its cell surface targets might be an advantage over a very high binding candidate in line with a previous EpCAM monoclonal antibody clinical trials for targeted cancer treatment. In this case, the high-affinity EpCAM monoclonal antibodies (*K*_d_ from 0.16 nM to 0.19 nM) caused acute pancreatitis in patients [25,26], whereas a particular EpCAM antibody with a medium-high affinity (*K*_d_ = 91 nM) showed better safety profile and well-tolerated in patients [27]. This was believed to be due to the ubiquitous expression of EpCAM protein, which is highly expressed in most human adenocarcinomas, however, it is also expressed at low levels in various normal epithelial cells [27]. As a result, although the tested high-affinity EpCAM antibody effectively targeted EpCAM over-expressed cancer cells, it also detrimentally targeted normal tissues expressing EpCAM at low levels. This could be true for EGFR. Although EGFR is overexpressed in certain cancer cells, it is also expressed in a number of normal cells and is essential for ductal development of the mammary glands [28]. Based on this, CL-4RNV616, with a moderate affinity (*K*_d_ = ~100 nM), could selectively interact with EGFR overexpressed cancer tissues. The specific recognition of the CL-4RNV616 aptamer to cell surface EGFR protein was further confirmed by fluorescence microscopy. As shown in Figure 5, following EGFR aptamer incubation, efficient binding (strong fluorescent signal) was observed in all of the three tested EGFR positive cells, whereas the EGFR negative HEK293 cells did not show any noticeable fluorescence signal. 

### 2.4. CL-4RNV616 Aptamer Initiated Strong Cytotoxicity in EGFR Positive Cancer Cells

**Figure 6:**
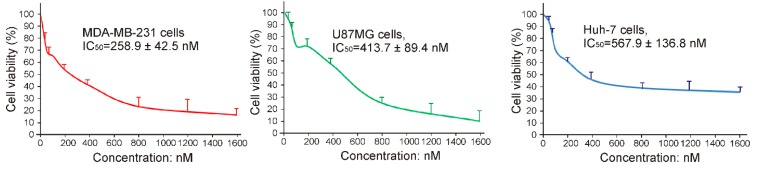
Evaluation of cytotoxicity and IC50 of CL-4RNV616 using EGFR positive Huh-7, MDA-MB-231, and U87MG cells.

**Figure 7:**
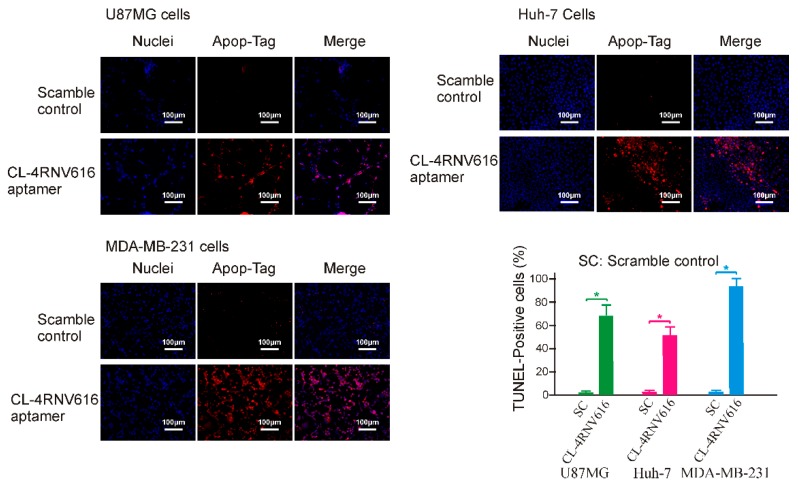
Evaluation of apoptotic induction effect of CL-4RNV616 aptamer on EGFR positive cancer cells. Representative micrograph of TUNEL assays on indicated cells after 72 h of treatment (at 500 nM). Blue, Hoechst 33342 (nuclei) and red, Rhodamine for apopTag-positive nuclei. The relative quantification was determined via the Image J program. * *p* < 0.05.

To investigate whether the newly developed 27mer CL-4RNV616 aptamer could inhibit cancer cell proliferation upon binding to EGFR, cytotoxicity assay was conducted using the EGFR positive U87MG, Huh-7, and MDA-MB-231cell lines. As shown in Figure S2, compared with the scrambled control group, 500 nM of CL-4RNV616 aptamer treatment significantly reduced cell viability in all of the tested cancer cells, whereas this was not observed in EGFR negative HEK293 cells. Further analysis to determine the half-maximal inhibitory concentration (IC50) revealed that CL-4RNV616 aptamer was effective in inhibiting cancer cell proliferation in a dose-dependent manner with an IC50 value of 258.9 nM for MDA-MB-231, 413.7 nM for U87MG, and 567.9 nM for Huh-7 cells respectively (Figure 6). To gain more insights on the anticancer potential of CL-4RNV616 aptamer, an apoptosis assay (TUNEL assay) was carried out using EGFR positive U87MG, Huh-7, and MDA-MB-231cell lines. As shown in Figure 7, after 72 h incubation with 500 nM of CL-4RNV616 aptamer, significant apoptosis was observed in all of the tested cancer cells. Compared to 2–3% apoptosis rate of the scramble control group, 63.4%, 51.8%, and 87.6% apoptosis rate were recorded in U87MG, Huh-7 and MDA-MB-231cells respectively, comparable to the 70.2% (U87MG), 53.4% (Huh-7), and 80.6% (MDA-MB-231) apoptosis rate induced by the original CL-4 aptamer (Figure S3). These data indicate that CL-4RNV616 aptamer-mediated inhibition of cell growth could be due to apoptosis activation effect, which is consistent with the previous observations with the original CL-4 aptamer in breast cancer [29] and lung cancer [16] cells.

### 2.5. CL-4RNV616 Aptamer Was Eligible for Clinical Cancer Diagnosis 

**Figure 8:**
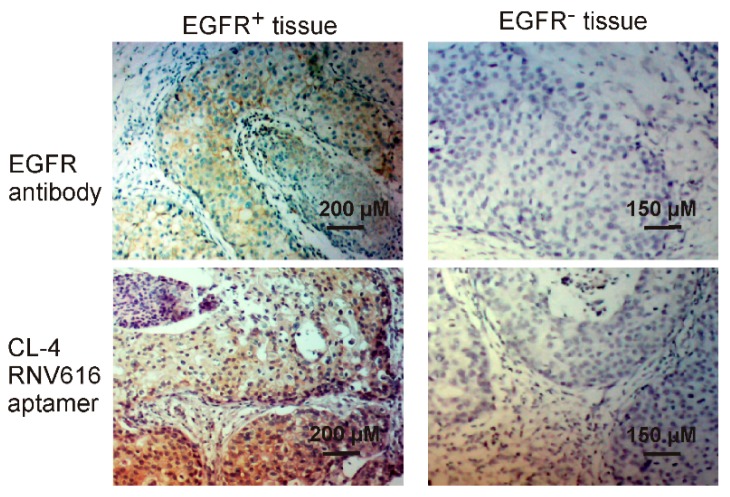
Tissue immunostaining of both EGFR positive and negative breast cancer by CL-4RNV616 aptamer and EGFR antibody. H&E stain was conducted for morphological confirmation.

Antibody-based tissue immunostaining plays an important role in current cancer diagnosis [30]. However, the application of antibodies is compromised by their low stability and higher production and purchase costs [31]. Aptamers, often termed as chemical antibodies- possess certain advantages over antibodies such as low batch-to-batch variation, reduced production cost, prolonged shelf life and ability to incorporate various chemical modifications for enhanced binding capacity and stability [19,23], which make them ideal alternatives for antibodies in immunohistochemistry. In this work, we extended the application of the developed CL-4RNV616 aptamer to detect EGFR positive cancer using clinical breast cancer biopsies. As shown in Figure 8, similar with the commercial EGFR antibody control, CL-4RNV616 aptamer effectively recognized cancer nests of the EGFR positive breast tumor sections and did not bind to EGFR negative breast tumor sections as well as background cells within tumor sites. These results may validate the scope of CL-4RNV616 aptamer as a probe for EGFR detection in clinical cancer sections. In this work, although only paraffin-embedded breast biopsies were tested, considering the efficient binding capacity of CL-4RNV616 aptamer to EGFR proteins on living cells (Figure 4 and Figure 5), the application of this aptamer may also be extended to immunostaining of other cancer tissues for helping towards cancer detection. 

In conclusion, we have developed a robust 27mer mixmer nucleic acid aptamer CL-4RNV616 containing 2′-OMe and DNA nucleotides which can efficiently target EGFR protein and maintain very high stability in human serum. CL-4RNV616 aptamer was very effective in targeting EGFR-overexpressed cancer cells and in addition, also demonstrated efficient inhibition and apoptosis of breast cancer, liver cancer and malignant glioblastoma cells in vitro. Although the in vivo efficacy of CL-4RNV616 aptamer is yet to be determined, based on our findings, we firmly believe that CL-4RNV616 could be used to target EGFR within in vitro assays at low cost.

## 3. Materials and Methods 

### 3.1. Buffers and Sequence Information

Binding buffer: Serum-free DMEM (ThermoFisher, Waltham, MA, USA, 12491-015) containing 100 µg/mL tRNA (Sigma, Darmstadt, German, R8759), Wash buffer: serum-free DMEM medium. All truncated aptamer sequences were synthesized in house on GE AKTA oligopilot plus 10 (GE Healthcare Life Sciences, Pittsburgh, PA, USA) oligonucleotide synthesizer using standard phosphoramidite chemistry in 1µmol scale. All synthesis reagents were purchased from Merck Millipore. The sequence information of CL-4 and CL-4RNV615—CL4-RNV618 as listed in Table 1. The scramble control sequence is 5′mCmGmUmUmAmUmAmGmUmUmAmAmGmGmCmGmUmGmUmGmCmCmGmUmCmAmU3′ (see Figure S4 for the HPLC analysis data of CL-4, CL-4RNV615 and CL-4RNV616). Before each treatment, the aptamers were denatured at 95 °C for 5 min and then snap cooled on ice for 10 min. EGFR protein was purchased from Sino Biotechnology (Wayne, USA, 10001-H08H). For imaging and ELONA assays, aptamers were synthesized with 5′-FAM and 5′-Biotin molecules. 

### 3.2. Truncation of Aptamer 

The original 39mer CL-4 aptamer was truncated to 27mer in length based on the predicted secondary structure using Mfold (http://unafold.rna.albany.edu/?q=mfold/rna-folding-form, access date: 30 July 2017) and RNAfold (http://rna.tbi.univie.ac.at/cgi-bin/RNAWebSuite/RNAfold.cgi, access date: 30 July 2017) web server platforms) [17,18] and synthesized as full 2′-OMe (CL-4RNV615), 2′-OMe/DNA mixmer (CL-4RNV616), full RNA (CL-4RNV617), and full DNA (CL-4RNV618) sequences (Table 1). 

### 3.3. Cell Culture 

U87MG (human glioblastoma) and HEK293 cells were purchased from Cell Bank Australia (Sydney, Australia). MDA-MB-231 cells were purchased from ATCC, USA, and kindly provided by A/Prof. Stacey Edwards at the Queensland Berghofer Institute for Medical Research. Huh-7 cells were kindly provided by MTL laboratory headed by Prof. Sue Fletcher and Prof. Steve Wilton at Murdoch University. All cells were cultured at 37 °C in Dulbecco’s Modified Eagle Media (ThermoFisher, USA, 12491-015) supplemented with 10% FBS (Sigma, F8192) and supplying 5% CO_2_/air. 

### 3.4. ELONA Assay

This experiment was performed using a similar procedure as previously reported [23]. Briefly, 1 µg of EGFR protein was added to 100 µL PBS and incubated with wells of the Maleic Anhydride Activated Plate (ThermoFisher, Waltham, USA, 15100) at room temperature for 1 h. Then, the wells were blocked with 100 µg/mL of yeast tRNA (Sigma, R8759) and 1% BSA (Sigma, Darmstadt, German, A2153) at room temperature for 1 h. After thorough washing with PBS, wells were incubated with 100 µL of 100 nM aptamers for 1 h at RT. Then the wells were washed with wash buffer followed by the incubation of HRP-conjugated anti-Biotin antibody (Alpha Diagnostic International, San Antonio, USA, 20361) for 1 h. Then, after thorough washing, the fluorescence intensity was determined with a FLUOstar Omega Microplate Reader (BMG LABTECH, Ortenberg, Germany) after the addition of QuantaBlu Fluorogenic Peroxidase Substrate (ThermoFisher, Waltham, MA, USA, 15169). 

### 3.5. TUNEL Apoptosis Assay 

Apoptosis was assessed using ApopTag Red In Situ Apoptosis Detection Kit (Millipore, Darmstadt, Germany, S-7165) in accordance with the manufacturers’ instructions. Briefly, cells were seeded in an 8-chamber slide (Lab-Tek, Campbell, PM, USA, 177402) at 20,000 cells/chamber 24 h before the test. After incubation, cells were treated with 500 nM of either CL-4 or CL-4RNV616 aptamer in DMEM medium for 72 h. After PBS washing, the cells were then fixed in 1% paraformaldehyde in PBS for 10 min at room temperature followed thorough wash with PBS. Then, the slide was incubated with 55 μL/5 cm^2^ of working strength TdT enzyme at 37 °C for 1 h. One hour later, the reaction was stopped and washed with stop/wash buffer at room temperature for 10 min. After thorough washing in PBS, the sections were then incubated with rhodamine-conjugated anti-digoxigenin antibody (1:50) at room temperature in a darkened humidified chamber for 30 min. After another round of thorough washing with PBS, the sections were counterstained with Anti-fade mounting medium (ThermoFisher, Waltham, USA, P36930) containing 3 μg/mL Hoechst 33342 (Sigma, Darmstadt, German, B2261) before visualization under a Nikon Eclipse TS100 Inverted Fluorescence Microscope system (Nikon, Tokyo, Japan). 

### 3.6. Fluorescence Imaging 

The aptamer-mediated cell binding was carried out as previously reported [20]. One hour after incubation with indicated aptamers at 200 nM concentration in wells of a 24-well plate, 1 µL of Hoechst 33342 solution (1 µg/mL) was added to the plated cells and incubated for 10min at 37 °C in a humidified incubator supplying 5% CO_2_/air. The cells were then washed prior to visualization using a Nikon Eclipse TS100 Inverted Fluorescence Microscope system (Nikon). 

### 3.7. Cell Viability Assay

MTT assay was conducted as previously reported [32]. Briefly, cells (3 × 10^3^ cells/well) in 200 µL of indicated culture mediums were seeded in 96-well plates and incubated for 24 h. After that, the culture medium was replaced by medium containing CL-4RNV616 aptamer at 12.5 nM, 25 nM, 50 nM, 100 nM, 200 nM, 400 nM, 800 nM, 1600 nM concentrations. After 48h incubation, 5 mg/mL MTT reagent (Sigma, M5655) in 1× PBS (20 µL/well) was added into the plates and incubated for 3 h. After incubation, the medium was aspirated and dimethyl sulfoxide (150 µL/well) was added to stop the reaction. The absorbance was quantified by a FLUROstar Omega multi-detection microplate reader (BMG Labtech, Ortenberg, Germany) at 570 nm wavelength. The cell viability was calculated by comparing the luminescent signal of treatment groups to the signal obtained with untreated cells (setting as 100% viability). Each value represents the mean standard deviation from triplicates. 

### 3.8. Flow Cytometry Assay 

Cells were first incubated with blocking buffer for 1 h on ice (binding buffer plus 100 µg/mL tRNA and 1% BSA) followed by two washes with binding buffer prior to incubation with serial concentrations of FAM-labelled aptamer in a 200 µL volume of binding buffer for 1 h at room temperature. The cells were then centrifuged, washed with binding buffer, and resuspended in 200 µL binding buffer and subjected to flow cytometry analyses (Beckman Coulter Gallios, Indianapolis, IN, USA). The mean fluorescence intensity (MFI) of the untreated cells was subtracted from that of the aptamer treated cells to generate the MFI of specific binding.

### 3.9. Determination of Aptamer Affinity

The equilibrium dissociation constant (K’d) of aptamers was determined according to a previous publication [19]. After either ELONA or Flow cytometry assay, the mean fluorescence intensity (for FACS assay) of the untreated group (background signal) was subtracted from that of the aptamer-target groups to generate the MFI of specific binding. Then the MFI of individual concentration groups was normalized with the concentration group displaying the highest MFI (set as 1.0). The equilibrium dissociation constant was calculated via GraphPad Prism 8 (GraphPad, San Diego, CA, USA). 

### 3.10. Immunohistochemistry Assay 

Fixation of breast cancer biopsy sections using acetone (Sigma, Darmstadt, German, V800023) for 30 s, followed by blocking with 0.1 mg/mL tRNA and 10% goat serum in DMEM medium for 1 h at room temperature. After removing the blocking buffer, the slides were then incubated in 200 μL of 100nM Biotin labelled aptamer or 1:1000 diluted anti-EGFR antibody (Abcam, San Francisco, CA, USA, ab52894) for 30 min at room temperature. Then, the sections were washed 3 times using serum-free DMEM medium, 5 mL per wash in a coplin jar. HRP conjugated anti-Biotin antibody (Abcam, ab19221) was then used to incubate sections at a concentration of 1:300 in DMEM medium at room temperature for 1 h. Then, the sections were subjected to DAB (Vector, Burlingame, CA, USA, SK-4100) development at room temperature for 5min, followed by thorough washing. The nucleus stain was conducted using Harris hematoxylin solution (Vector, Burlingame, USA, H-3502) for 5 min at room temperature. After that, the sections were differentiated in 1% acid alcohol for 5 s, and bluing in Scott’s tap water substitute (Sigma, Darmstadt, German, S5134) for 1 min. The sections were then dehydrated in 70%, 95% and absolute alcohol for 2 min each. Finally, the sections were mounted with histolene based mounting medium (Sigma, Darmstadt, German, H2779) and forwarded to imaging. Breast cancer biopsies were obtained from consenting patients through the First Affiliated Hospital of Dalian Medical University (Dalian, China). Application of clinical breast cancer samples and related protocols have been approved by the ethics committee of the Dalian Medical University, No: SCXK (Liao) 2017-0129].

### 3.11. Statistical Analysis 

All statistical analyses were performed using GraphPad Prism 8.0. An unpaired *t*-test was used for comparisons between two experimental groups, and ANOVA was used for comparisons of more than two groups. Unless otherwise indicated, all results were averaged from biological triplicates and values are reported as means ± SEM. A P value of less than 0.05 was considered statistically significant. 

## Figures and Tables

**Table 1:** Sequences of the truncated and chemically-modified epidermal growth factor receptor (EGFR) aptamers.

Name	Sequence (5′–3′) & Size (nt)
CL-4	rGfCfCfUfUrArGfUrArAfCrGfUrGfCfUfUfUrGrAfUrGfUfCrGrAfUfUfCrGrAfCrArGrGrArGrGfC (39)
CL4 RNV615	m(UGCUUUGAUGUCGAUUCGACAGGAGGC) (27)
CL-4 RNV616	mUmGmCmUmUmUmGdAmUmGmUmCmGdAmUmUmCmGdAmCdAmGmGdAmGmGmC (27)
CL-4 RNV617	r(UGCUUUGAUGUCGAUUCGACAGGAGGC) (27)
CL-4 RNV618	d(TGCTTTGATGTCGATTCGACAGGAGGC) (27)

Note: RNA nucleotides are represented by ‘r’, 2′-Fluoro nucleotides are represented by ‘f’, 2′-O-Methyl nucleotides are represented by ‘m’ and DNA nucleotides are represented by ‘d’.

## Supplementary Materials

Supplementary materials can be found at https://www.mdpi.com/1422-0067/20/19/4700/s1. Figure S1: Predicted secondary structure of CL-4 aptamer and the 27mer truncated variant; Figure S2: CL-4RNV616 aptamer specifically reduced the proliferation of EGFR positive cancer cells. EGFR positive Huh-7, MDA-MB-231, U87MG cells, as well as EGFR negative HEK293 cells were incubated with CL-4RNV616 aptamer at 500 nM concentrations for 72 h. The cell viability was determined via a MTT assay; Figure S3: Evaluation of apoptotic induction effect of CL-4 aptamer on EGFR positive cancer cells. Representative micrograph of TUNEL assays on indicated cells after 72 h of treatment (at 500 nM). Blue, Hoechst 33342 (nuclei) and red, Rhodamine for apopTag-positive nuclei. The relative quantification was determined via Image J program; Figure S4: Ion Exchange-HPLC analysis of CL-4RNV615, CL-4.

## Abbreviations

EGFR	Epidermal growth factor receptor
MFI	Mean fluorescence intensity
TUNEL	Terminal deoxynucleotidyl transferase dUTP nick end labeling
