# Supplementary material for: Efficient Epidermal Growth Factor Receptor Targeting Oligonucleotide as a Potential Molecule for Targeted Cancer Therapy
Source: Int J Mol Sci. 2019 Sep 22;20(19):4700. doi: 10.3390/ijms20194700 (PMC6801465; doi:10.3390/ijms20194700)
Supplement: Supplementary file 1 [file ijms-20-04700-s001.pdf]

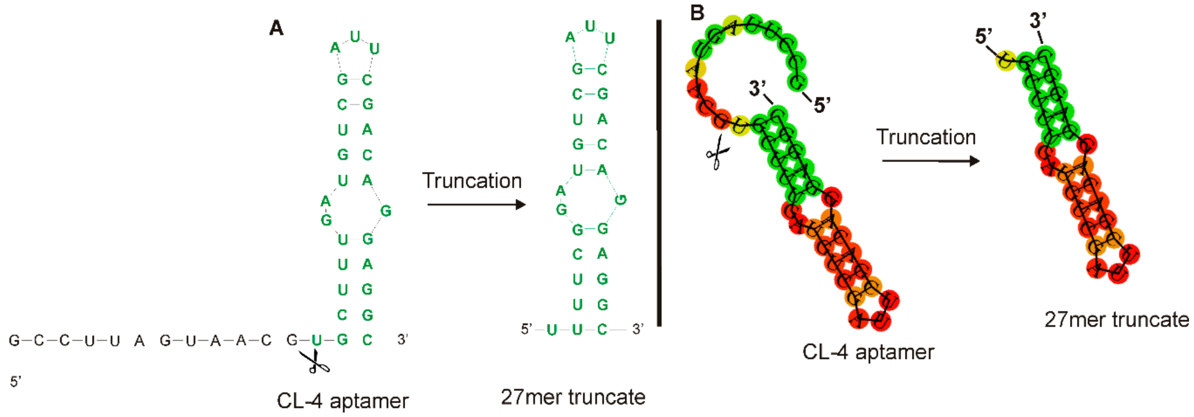

Figure 1. Predicted secondary structure of CL-4 aptamer and the 27mer truncated variant.

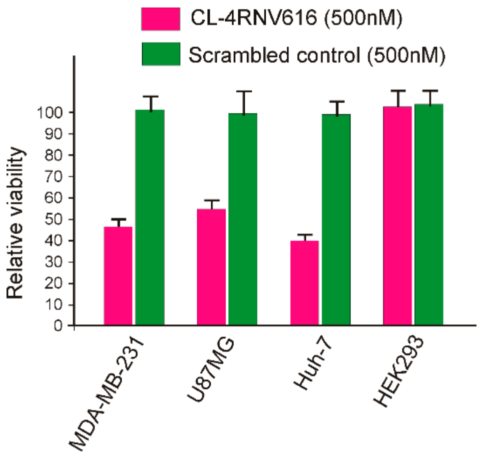

Figure S2. CL-4RNV616 aptamer specifically reduced the proliferation of EGFR positive cancer cells. EGFR positive Huh-7, MDA-MB-231, U87MG cells, as well as EGFR negative HEK293 cells were incubated with CL-4RNV616 aptamer at 500 nM concentrations for 72 h. The cell viability was determined via a MTT assay.

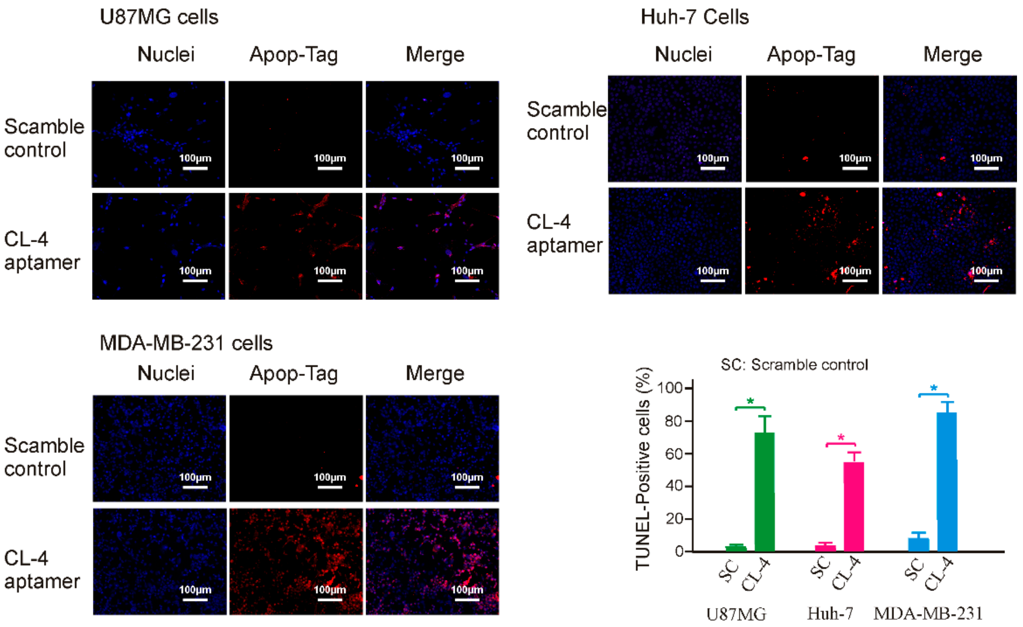

Figure S3. Evaluation of apoptotic induction effect of CL-4 aptamer on EGFR positive cancer cells. Representative micrograph of TUNEL assays on indicated cells after 72 h of treatment (at 500 nM).

Blue, Hoechst 33342 (nuclei) and red, Rhodamine for apopTag-positive nuclei. The relative quantification was determined via Image J program.

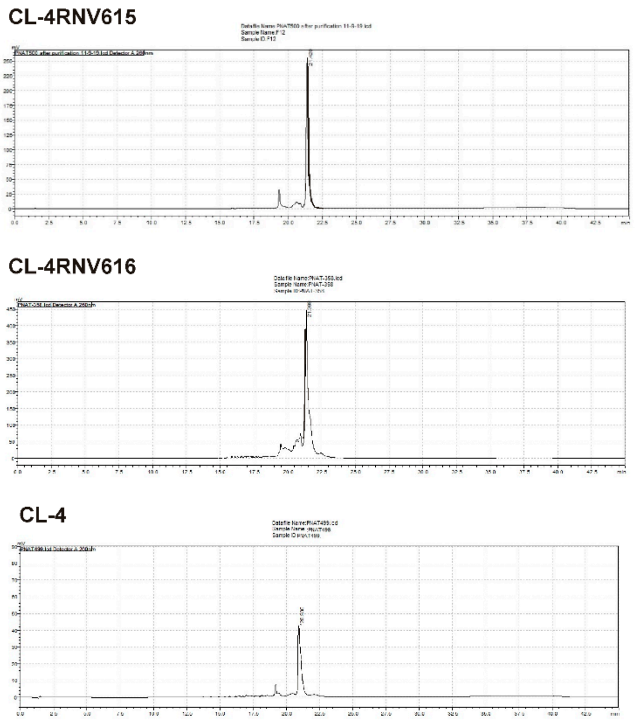

Figure S4. Ion Exchange-HPLC analysis of CL-4RNV615, CL-4.
